# Ferromagnetic Properties of N-Doped and Undoped TiO_2_ Rutile Single-Crystal Wafers with Addition of Tungsten Trioxide

**DOI:** 10.3390/ma11101934

**Published:** 2018-10-11

**Authors:** Jing Xu, Haiying Wang, Zhongpo Zhou, Zhaorui Zou

**Affiliations:** 1 College of Physics and Materials Science, Henan Normal University, Xinxiang 453007, China; xujinghenan@126.com (J.X.); paul@whu.edu.cn (Z.Zh.); zrzou@whu.edu.cn (Z.Zo.); 2 School of Physics and Technology, Wuhan University, Wuhan 430072, China

**Keywords:** ferromagnetic properties, oxygen vacancy, WO_3_-loaded, rutile TiO_2_ single-crystal wafers

## Abstract

In this work, undoped, N-doped, WO_3_-loaded undoped, and WO_3_-loaded with N-doped TiO_2_ rutile single-crystal wafers were fabricated by direct current (DC) magnetron sputtering. N-doping into TiO_2_ and WO_3_ loading onto TiO_2_ surface were used to increase and decrease oxygen vacancies. Various measurements were conducted to analyze the structural and magnetic properties of the samples. X-ray diffraction results showed that the N-doping and WO_3_ loading did not change the phase of all samples. X-ray photoelectron spectroscopy results revealed that W element loaded onto rutile single-crystal wafers existed in the form of WO_3_. UV-Vis spectrometer results showed that the absorption edge of WO_3_-loaded undoped and WO_3_-loaded with N-doped TiO_2_ rutile single-crystal wafers had red shift, resulting in a slight decrease in the corresponding band gap. Photoluminescence spectra indicated that oxygen vacancies existed in all samples due to the postannealing atmosphere, and oxygen vacancies density increased with N-doping, while decreasing with WO_3_ loading onto TiO_2_ surface. The magnetic properties of the samples were investigated, and the saturation magnetization values were in the order N-doped > WO_3_-loaded with N-doped > undoped > WO_3_-loaded undoped rutile single-crystal wafers, which was the same order as the oxygen vacancy densities of these samples. N-doping improved the saturation magnetization values, while WO_3_-loaded decreased the saturation magnetization values. This paper reveals that the magnetic properties of WO_3_-loaded with N-doped rutile single-crystal wafers originate from oxygen vacancies.

## 1. Introduction

Since 1998, when Prinz [1] first found novel applications of dilute magnetic semiconductors (DMSs) in spintronic devices, such as spin light-emitting diode, magnetic random access memory, and spin field emission transistor [2,3], a growing number of researchers have studied the ferromagnetic behavior of DMSs and the origin of this ferromagnetism. Many relevant experiments have been successfully conducted to study the room temperature ferromagnetic behavior of DMSs, especially TiO_2_-based DMSs [4,5,6,7]. Researchers studying TiO_2_-based DMSs ferromagnetism have mainly concentrated on element doping, such as Co, Mn, Ru, Ni, Fe, Cu, Sm, S, N [8,9,10,11,12,13,14,15,16,17], and so on. Based on these studies, several models have emerged to illustrate DMSs’ room temperature ferromagnetic origin, such as the Stoner-type model [18], the band-coupling model [19], and the Ruderman–Kittel–Kasuya–Yosida model. Furthermore, researchers have also recently proposed oxygen vacancies as the origin of room temperature ferromagnetism (RTFM) [6,20,21]. The true origin of RTFM is therefore difficult to find. Wang et al. [22] suggested that RTFM in nitrogen-doped rutile TiO_2_ powders are rooted on oxygen vacancies (Vos) induced by N-doping. As N-doping is an effective method to produce Vos, it is known that WO_3_ is a very stable metal oxide [23] and can protect TiO_2_ wafer surface after loading onto it. In this work, we selected WO_3_ loading and N-doping method to adjust and control Vos densities in TiO_2_ wafers to study the origin of TiO_2_’s room temperature ferromagnetic behavior.

Undoped, N-doped, WO_3_-loaded undoped, and WO_3_-loaded with N-doped TiO_2_ rutile single-crystal wafers were fabricated by magnetron sputtering. The magnetic properties and optical properties of the prepared samples were studied. The largest saturation magnetization (Ms) values were found in N-doped rutile single-crystal wafers. The Ms values were adjusted by N-doping and WO_3_ loading. The N-doping increased the Ms values by increasing Vos density, while WO_3_-loading decreased the Ms values by decreasing Vos density. The room temperature ferromagnetic property was adjusted by controlling the Vos.

## 2. Materials and Methods

TiO_2_ rutile single-crystal wafers oriented (100) (named as sample 1) in the size of 5 mm × 5 mm × 0.5 mm were used as substrates, which were bought from Beijing Scistar Technology Co., Ltd. (Beijing, China). The N-doped TiO_2_ single-crystal wafers (named as sample 2) were obtained by annealing sample 1 in N_2_ and NH_3_ mixed atmosphere at 500 °C for 2 h. WO_3_-loaded TiO_2_ single-crystal wafers (named as sample 3) were obtained by wafers grown on TiO_2_ substrates by direct current (DC) magnetron sputtering with a W target in a small ion sputter apparatus (Cressington sputter coater 108) at room temperature. After loading the W element, samples were postannealed in air at 450 °C for 2 h to get sample 3. Following the N-doping process for sample 3, WO_3_-loaded with N-doped TiO_2_ single-crystal wafers (named as sample 4) were obtained. The structural properties of the samples were characterized by X-ray diffraction (XRD, Bruker D8 Discover, Karlsruhe, Germany) with 2θ scanning range from 20° to 80°, and the morphology of the samples was observed by scanning electron microscopy (SEM, S-4800, Hicathi, Tokyo, Japan). The surface element composition and chemical valence states were measured with X-ray photoelectron spectroscopy (XPS, ESCALAB 250Xi, Thermo Fisher Scientific, Waltham, MA, USA). The optical properties and band gap were studied using the absorption spectra from a UV-Vis spectrometer (UV 3600, Shimadzu, Kyoto, Japan). The photoluminescence (PL) spectra were obtained by He–Cd laser (Persee, Beijing, China) with 280 nm wavelength of the excitation light source. The magnetic properties of samples were characterized at room temperature by vibrating sample magnetometer (VSM, Quantum Design PPMS-9, San Diego, CA, USA) with a magnetic field from −8 K Oe to 8 K Oe. 

## 3. Results and Discussion

**Figure 1:**
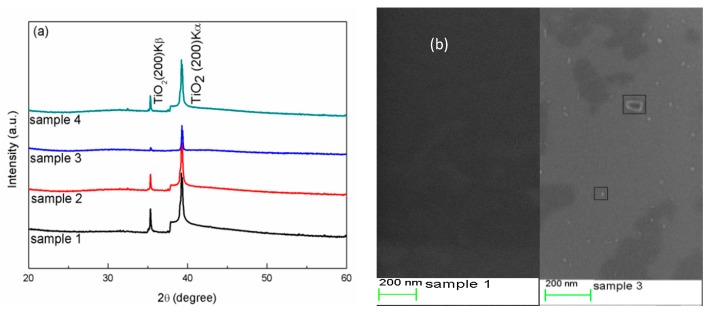
(**a**) XRD spectra of samples 1–4 prepared by magnetron sputtering. (**b**) The top view of SEM microphotograph of sample 1 (**left**) and sample 3 (**right**).

The crystal structures of the samples were characterized by XRD, and the XRD patterns of all samples are shown in Figure 1a (the Y-axis is a logarithmic scale). As can be seen in the figure, a primary peak appeared at 39.16°, which corresponded to the TiO_2_ rutile (200) plane, while a weak peak appeared at 35.18° corresponding to the TiO_2_ rutile (200) K_β_ [24]. Only (200) diffraction peaks were detected in all samples. Therefore, all samples were mainly oriented in the (100) direction. The peak intensities of sample 3 were weaker than those of other samples, but the relative intensities were the same as other samples. The XRD patterns had no peaks of products for tungsten and tungsten oxide due to the low sputtering concentration of WO_3_. These results were similar to Sajjad et al.’s. report, where no peaks related to W element were observed even with a W sputtering concentration up to 4% [25]. The content of W element was 2.17% in our experiment, which was determined by XPS measurements.

The surface morphology of samples 1 and 3 are shown in Figure 1b. From sample 3, it was obvious that many ash-colored particles were loaded on the sample surface compared to sample 1, where the surface was smooth and clean. The diameters of the deposited particles varied from 10 to 80 nm. The deposited particles might be related to W oxide in the special process of forming sample 3.

**Figure 2:**
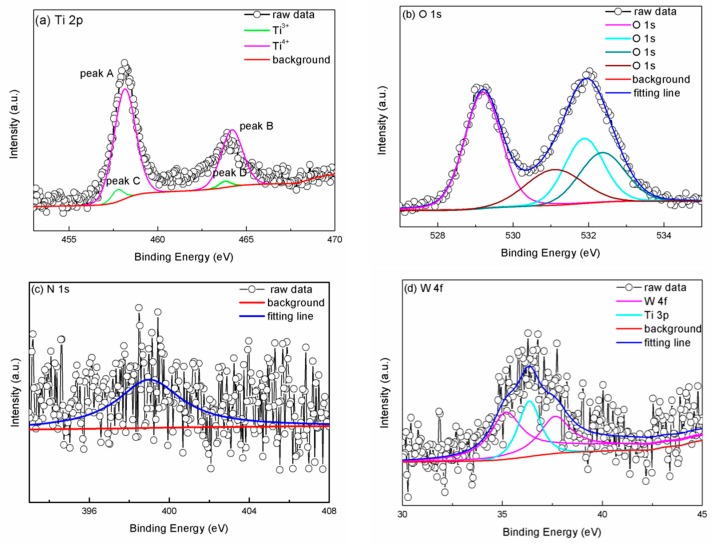
XPS spectra for (**a**) Ti 2p, (**b**) O 1s, (**c**) N 1s, and (**d**) W 4f core-level for sample 4, respectively.

To verify the surface elemental composition and elemental chemical valence state, XPS measurements were conducted for sample 4. The binding energy (BE) of all peaks in spectral line was calibrated by C 1s at 284.8 eV. Figure 2a–d displays Ti 2p, O 1s, N 1s, and W 4f core-level spectra for sample 4, respectively. It is known that there are some rules in peak splitting of XPS results: (i) The intensity ratio between the doublets of p, d, and f atomic orbital should be 2:1, 3:2, and 4:3, respectively. (ii) The full width at half maximum (FWHM) values of doublets in the same valence state of the same element should be equal. (iii) The FWHM of the high valence state of the same element should be greater than or equal to the FWHM of low valence state. All spectral lines in our experiment were fitted with Gaussian–Lorentzian function. The red lines were the background, and the lines with symbols were the original experiment data. As can be seen in Figure 2a, peak A and peak B appeared at BE of 458.2 eV and 464.2 eV, respectively, which corresponded to Ti^4+^ standard peak according to the handbook of XPS [26], and two weak peaks appeared at BE of 457.7 eV and 463.8 eV. The BE of Ti^2+^ in TiO appears at around 455 eV and Ti^4+^ in TiO_2_ appears at around 459 eV, according to the XPS handbook, which means that Peak C and peak D corresponded to the Ti^3+^ standard peak. The fitted doublets of Ti^4+^ and Ti^3+^ met the aforementioned peak splitting rules. The XPS result of Ti meant that little of Ti^3+^ coexisted with Ti^4+^ states in sample 4. As can be seen in Figure 2b, there were four O 1s peaks, meaning there were four different chemical environments in sample 4. A relatively strong peak appeared at BE of 529.20 eV, which was attributed to lattice oxygen in TiO_2_ [27,28]. A small peak that appeared at 531.9 eV corresponded to Ti-N-O band in sample 4 due to the doping of N element in the sample; this finding is in agreement with Chan and Lu’s report, which showed that the BE of Ti-N-O band is 531.5 eV [29]. One weak peak appeared at BE of 532.3 eV, which could be ascribed to the C=O band and the hydroxyl groups due to the contamination of the sample surface [30,31]. Another peak appeared at BE of 531.1 eV, which could be attributed to the W-O band in WO_3_ [32,33]. As can be seen in Figure 2c, a weak peak was observed at 399.02 eV, which could be attributed to N in the Ti-N-O group [34]. The N 1s peak intensity was apparently not high because the NH_3_ content was only 5% in the N_2_/NH_3_ atmosphere in the experimental procedure. In Figure 2d, it can be seen that the peak of purple spectral line appeared at BE of 35.1 eV and 37.7 eV, which could be attributed to W 4f 7/2 and 5/2 doublets in WO_3_, respectively. The distance between the doublets was 2.5 eV, which was close to the standard distance of 2.15 eV [26]. The fitted doublets of W 4f met the aforementioned peak splitting rules. Besides, a peak appeared at BE of 36.3 eV. In Yamashita and Hayes’ report, the BE of Ti 3p appeared at 36.0 eV inTi_2_O_3_ and 37.5 eV in TiO_2_ [35]. This meant that the peak that appeared at BE of 36.3 eV could be assigned as Ti 3p. The result verified the existence of Ti^4+^ and Ti^3+^ in the sample, consistent with previous analysis. The XPS spectra result of W 4f core level indicated that W element existed on the surface of WO_3_-loaded with N-doped samples in the form of +6 chemical valence states. This means that WO_3_ was loaded on the TiO_2_ sample surface rather than impregnated into the TiO_2_ lattice. The loading of WO_3_ on the wafer surface did not alter the chemical state of the Ti or O element of the wafer.

**Figure 3:**
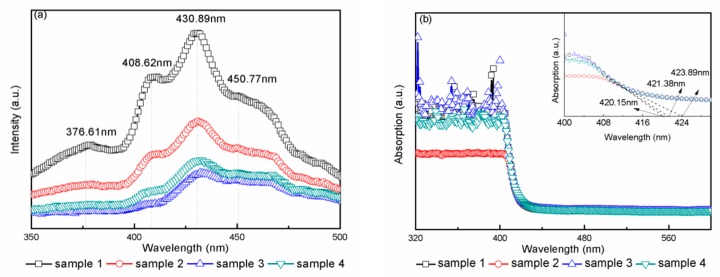
(**a**) Photoluminescence (PL) spectra and (**b**) absorption spectra for samples 1–4. The insert in (**b**) shows a zoomed-in view of the surrounding of the absorption edge.

To investigate the electron–hole recombination rate and the existence of Vos, PL spectra of all samples were collected at the wavelength range of 350–500 nm and are shown in Figure 3a. In the wavelengths from 350 to 410 nm, all samples had two emission peaks, which were attributed to band edge emission. The emission peak position at 376.61 nm originated from charge recombination at the shallow-trap surface states [36]. The emission peak appearing at 408.62 nm corresponded to a band gap transition from conduction band to valence band, which was equal to the band gap of rutile TiO_2_ (3.02 eV). The emission peak at 430.89 nm might have been due to the self-trapped excitons in the TiO_6_ octahedra [37]. The emission peak at 450.77 nm and 476 nm were related to Vos in the samples due to the recombination of photogenerated holes and electrons [38,39]. 

As for intensity, sample 1 had the strongest peak intensity compared to the other three samples. The weak PL intensity in sample 3 indicated the low radiative recombination rate of the electrons and holes. Photogenerated electrons were transfer from TiO_2_ conduction band to tungsten oxide conduction band, and the holes accumulated in the TiO_2_ valence band. As a result, photogenerated electrons and holes were effectively separated. In addition, when the tungsten oxide concentration was lower than its optimal ratio, the tungsten energy level would be a separation center [25]. All these reasons contributed to a low PL intensity in samples 3 and 4. The concentration of Vo in sample 2 increased due to the annealed atmosphere, the excited electrons were easily trapped by Vos, and the holes were trapped by the doped nitrogen atoms [40], leading to the separation of electrons and holes.

The optical properties and band gap of the prepared samples were investigated by UV-Vis spectrophotometer. Figure 3b displays the absorption spectra of samples 1–4. All samples had absorption edges ranging from 400 to 425 nm due to the band-to-band transition from the Ti 2p valence band to the conduction band in the TiO_2_ single crystals. The absorption edge of samples 1–4 was 420.15 nm, 423.89 nm, 420.15 nm, and 421.38 nm, respectively. It was obvious that samples 2 and 4 had red shift, which was similar to other reports [41,42]. The band gap was calculated by the equation: Eg = hc/λ, where c is the light velocity equal to 3 × 10^8^ m/s, h is Planck’s constant equal to 6.626 × 10^−34^ J s, λ is the corresponding wavelength of the samples, Eg is the calculated result (band gap) of the samples. The calculated band gap of samples 1–4 was 2.96 eV, 2.93 eV, 2.96 eV, and 2.95 eV, respectively. Samples 1 and 3 had the same band gap values, which was due to WO_3_ being simply deposited on the single-crystal wafers. Compared to sample 1, the band gap of samples 2 and 4 was slightly decreased, as in the report by Chen et al. [32]. 

**Figure 4:**
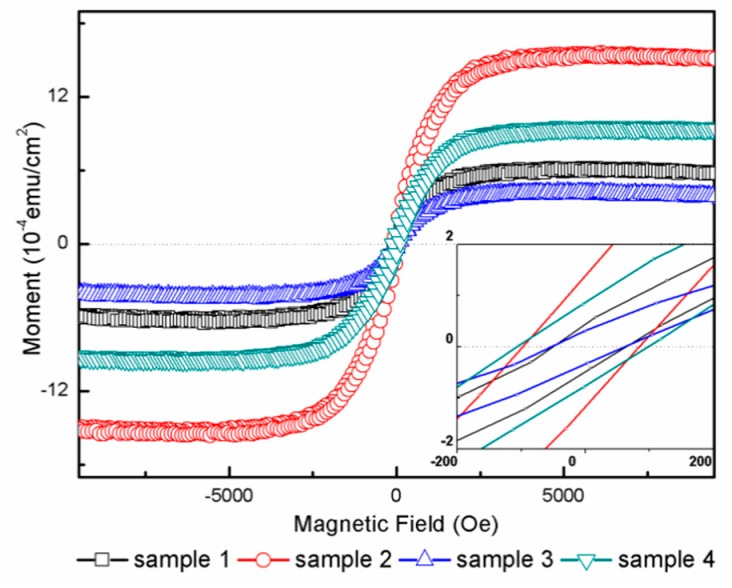
Magnetic field vs. moment curve of samples 1–4 measured at room temperature. The insert shows a zoomed-in view of the small field.

To further investigate the magnetic property of the samples, the magnetic moment vs. the magnetic field curves were collected at room temperature, as shown in Figure 4. The enlarged figure in the inset near zero magnetic field has been provided to distinctly illustrate the coercive force (Hc). The figure was plotted after deducting diamagnetic substrate. All the samples had a typical magnetic hysteresis loop, indicating that all samples were ferromagnetic materials. As can be seen in Figure 4, sample 2 yielded the highest Ms value (15.15 × 10^−4^ emu/cm^2^), while sample 3 had the lowest Ms value (4.16 × 10^−4^ emu/cm^2^). Sample 4 had a larger Ms value of 9.42 × 10^−4^ emu/cm^2^ than sample 1, which had a value of 6.05 × 10^−4^ emu/cm^2^. Furthermore, the Ms values were in the order of sample 2 > sample 4 > sample 1 > sample 3. The coercive force values (Hc) of samples 1–4 were 54.72 Oe, 98.50 Oe, 54.72 Oe, and 111.37 Oe, respectively. 

As in Kim et al.’s report [43], the undoped rutile single-crystal wafer had a small Ms in our experiment. In addition, Kim et al. reported that Vos induces a lattice distortion in rutile TiO_2_ and introduces ferromagnetism in undoped TiO_2_ wafers due to the charge distribution. Sample 2 had the largest Ms values compared to others due to the high oxygen vacancy concentration, which indicated that the Vos was the main source of ferromagnetic behavior. For samples 3 and 4, the WO_3_ loading on the wafer surface caused lower Ms compared to samples 1 and 2. The demagnetizing field could be formed due to polarization by the addition of WO_3_, resulting in an increase in magnetic loss [23]. Therefore, WO_3_ loading decreased the Ms values. 

In this study, WO_3_ did not show any ferromagnetism, which would rule out any ferromagnetic contribution related to the W second phase. The ferromagnetism of sample 3 must have been intrinsic and originated from the oxygen vacancy. For sample 4, the WO_3_ deposited on the sample’s surface formed the demagnetizing field, resulting in an increase in magnetic loss. Therefore, the WO_3_-loaded undoped samples had the lowest Ms values compared to the others. For N-doped samples, due to an increase in Vos, N-doped samples had the highest Ms values compared to others. Considering the existence of many Vos, the influence of Vos on ferromagnetism can be explained by the carrier-mediated bound magnetic polaron model (BMP). The ferromagnetic coupling between two Ti^3+^ ions was formed through Vos. When the Vos concentration exceeded a certain value, the bound magnetic polaron caused the ferromagnetic coupling [44,45,46]. The weak ferromagnetic property of sample 4 was due to the low concentration of oxygen as well as the defects of the nanoparticles loaded on the sample surface, which might have decreased ferromagnetic coupling. Briefly, the more Vos present in the samples, the more apparent was the ferromagnetic behavior [47]. 

## 4. Conclusions

In conclusion, undoped, N-doped, WO_3_-loaded undoped, and WO_3_-loaded with N-doped TiO_2_ rutile single-crystal wafers were prepared by magnetron sputtering. Oxygen vacancy was increased by N-doping into TiO_2_ single-crystal wafers. XPS results showed that W element existed in the form of WO_3_ loaded onto wafer surface, and PL spectra indicated that WO_3_ loaded onto wafers decreased oxygen vacancy concentration. The N-doped TiO_2_ rutile single-crystal wafers had the maximum magnetic saturation value. The magnetic properties of the samples were in accordance with the oxygen vacancy concentration, which meant that the magnetic properties of the samples were sensitive to oxygen vacancy. Oxygen vacancies play a dominant role in the origin of WO_3_ loaded, N-doped TiO_2_ rutile single-crystal wafers. This work provides a fundamental understanding of the origin of the ferromagnetic properties of DMSs, which is critical for the incorporation of DMSs in future device applications.
